# Cyclic Thrombocytopenia: A Rare Cause of Recurrent Thrombocytopenia

**DOI:** 10.7759/cureus.22525

**Published:** 2022-02-23

**Authors:** Mosunmoluwa Oyenuga, Afoma Onyechi, Sara Sartaj, Rushin Patel, Jyotsana Sinha

**Affiliations:** 1 Internal Medicine, SSM (Sisters of St. Mary) Health St. Mary's Hospital, St. Louis, USA; 2 Hematology and Medical Oncology, SSM (Sisters of St. Mary) Health St. Mary's Hospital, St. Louis, USA

**Keywords:** cyclical thrombocytopenia, cyclic thrombocytopenia, low platelet count, thrombocytopenia, ctp

## Abstract

Cyclic thrombocytopenia (CTP) is a very rare hematological disorder that is characterized by periodic fluctuations in platelet counts. Diagnosis is generally delayed due to its similarity with immune thrombocytopenia (ITP). The pathophysiology is unknown and there are currently no guidelines for management. Many patients are usually treated for ITP initially prior to diagnosis. We describe a 67-year-old female with a history of multiple episodes of transient thrombocytopenia who presented to the hospital with another episode of thrombocytopenia. Her workup including HIV, hepatitis screening, vitamin B12, and folate was negative. She received a unit of platelet transfusion and was later observed in the hospital. Further review of her chart showed similar episodes in the past with spontaneous improvement. She was diagnosed with CTP. Her platelet count improved remarkably prior to discharge. In patients with recurrent fluctuation in their platelet count, CTP should be one of the differentials as this might prevent further unnecessary therapies.

## Introduction

Cyclic thrombocytopenia (CTP) is a very rare hematological disorder in which patients have low and normal platelet counts at varying times [1]. It is characterized by fluctuations in the platelet count, a hallmark of the disease [2]. Patients are usually underdiagnosed or misdiagnosed due to its similarity with immune thrombocytopenia (ITP), a diagnosis of exclusion in which patients have low platelet counts less than the lower limit of normal. Many cases reported have been idiopathic and some have been related to underlying malignancy and thyroid diseases [3]. Currently, there are about 70 reported cases and are more common in women [2]. There is no guideline for management for these patients. We present a case of long-term recurrent fluctuations in platelet count and negative workup who was later diagnosed with CTP. 

## Case presentation

Our patient is a 67-year-old female with a past medical history significant for type 2 diabetes, hypertension, and hyperlipidemia. She also has a past surgical history significant for thyroid lobectomy. She presented to the hospital for further management after she was found to have a low platelet count of 5 x 10^9/L (range: 153-416 x10^9/L) on her blood work during her primary care physician clinic visit. On further history, she reported monthly petechiae in her extremities and she recently noticed new upper lip hyperpigmentation. On physical examination, she had petechiae on both upper and lower extremities and upper lip. There was no hepatosplenomegaly or lymphadenopathy. Laboratory findings revealed thrombocytopenia with platelet count of 5 x 10^9/L (range: 153-416 x10^9/L), normal white blood cell count of 6.8 x 10^9 (range: 4.4-10.7x10^9/L), normal hemoglobin of 12.5 gm/dL (range: 12.0-15.6 gm/dL), and microcytosis 75.5fl (range: 80.7-98.3 fl). Further workup including coagulation d-dimer panel, HIV test, hepatitis screening, haptoglobin, lactic dehydrogenase (LDH), vitamin B12, iron studies, and folate were normal. Pathology review of peripheral smear had no evidence of schistocytes, dyspoietic, myeloid elements, or blasts. However, platelets were decreased, morphologically unremarkable, and no clumping was noted. On account of her significant thrombocytopenia, she was given a unit of platelets, which was stopped due to anaphylaxis. On further review of laboratory workup in the past several years, she was noted to have multiple episodes of low platelet counts (Table 1).

**Table 1:** Platelet count over a period of time

Date	Platelet count (10^9/L)
07/13/2009	92
11/16/2009	31
11/30/2009	203
08/01/2011	255
03/11/2013	204
10/08/2013	6
10/09/2013	56
07/21/2014	293
08/31/2015	57
01/18/2016	200
08/01/2017	92
05/23/2018	204
07/23/2020	103
12/21/2021	5
12/22/2021	103
12/23/2021	97
12/29/2021	422

Laboratory workup at that time included hepatitis viral panel, antinuclear antibody (ANA) screening, lupus anticoagulant panel, a disintegrin and metalloproteinase with a thrombospondin type 1 motif, member 13 (ADAMTS 13) activity, and quantitative rheumatoid factor, which were all negative. Based on her recurrent pattern of fluctuations in her platelet count with improvements sometimes without intervention, she was diagnosed with CTP. During her hospital stay, she did not receive any further treatment for her thrombocytopenia. Her platelet count improved to 97,000/L prior to discharge. Repeat platelet check one week after showed a spontaneous increase in her platelet count to 422 x 10^9/L.

## Discussion

CTP is often missed in patients who present with thrombocytopenia and have mild to no symptoms. Due to its rarity, it is usually not a common differential in patients who present with thrombocytopenia, and patients are usually diagnosed late. ITP, a common cause of thrombocytopenia, is often diagnosed in these patients. ITP is a diagnosis of exclusion; with negative workup as seen in this case, these patients are usually managed as such, and the possibility of CTP is not considered. CTP, however, requires a more temporal evaluation of platelet counts, where the fluctuations in platelet counts are evident over time.

The pathophysiology of CTP remains unknown. Some mechanisms that have been reported include abnormalities in the bone marrow, increased platelet destruction, and impairment of the regulatory mechanism for the production of platelets [3,4]. Some have reported a relationship with the menstrual cycle though, in our patient, these fluctuations still occurred after menopause [5,6]. Laboratory and clinical features that have been observed in patients with CTP are generally heterogeneous. Some patients have related thyroid disorders or underlying malignancies [3]. Our patient had a history of thyroid lobectomy in the past and reported pathology findings were benign.

Currently, there are no guidelines for the treatment of CTP. Many patients are usually treated as ITP with steroids, rituximab, or splenectomy. Some of them have a rapid increase in platelets after initiation of ITP therapy, which is erroneously interpreted as a good response. In reality, the rise in platelets is not due to therapy but reflects the natural course of CTP. Invariably, platelet counts will soon drop, often to a low level, thereby mimicking treatment failure [3]. This can lead to exposure to further therapies that might cause adverse effects. Most experts have advocated for close monitoring of platelets counts as they increase spontaneously without treatment and sometimes resolve completely [3]. Patients with CTP usually have mild symptoms such as purpura, petechiae, epistaxis, gingival bleeding, menorrhagia, and easy bruising [7], though there is a case report of a patient with CTP who later died of hemorrhagic stroke [8]. Some therapies that have been used for the treatment of CTP include danazol (10mg/kg per day for six months was reported), thrombopoietin receptor agonists (such as romiplostim, eltrombopag), and cyclosporine A (200 mg ) with variable responses [9,10].

## Conclusions

In conclusion, our patient was diagnosed with CTP, which is a rare cause of recurrent thrombocytopenia. Cyclical fluctuations in the platelet count with spontaneous improvement without medications should raise awareness for CTP as a differential diagnosis when evaluating patients with thrombocytopenia. Clinicians should have a high index of suspicion in patients who are noted to have recurrent episodes even after initial treatment for possible ITP as some of these patients might have CTP, thereby preventing unnecessary treatment for patients.
